# Top-down effect of dialogue coherence on perceived speaker identity

**DOI:** 10.1038/s41598-023-30435-z

**Published:** 2023-03-01

**Authors:** Lena Warnke, Jan P. de Ruiter

**Affiliations:** 1 Department of Psychology, Tufts University, Medford, MA USA; 2 Department of Computer Science, Tufts University, Medford, MA USA

**Keywords:** Human behaviour, Psychology

## Abstract

A key mechanism in the comprehension of conversation is the ability for listeners to recognize who is speaking and when a speaker switch occurs. Some authors suggest that speaker change detection is accomplished through bottom-up mechanisms in which listeners draw on changes in the acoustic features of the auditory signal. Other accounts propose that speaker change detection involves drawing on top-down linguistic representations to identify who is speaking. The present study investigates these hypotheses experimentally by manipulating the pragmatic coherence of conversational utterances. In experiment 1, participants listened to pairs of utterances and had to indicate whether they heard the same or different speakers. Even though all utterances were spoken by the same speaker, our results show that when two segments of conversation are spoken by the same speaker but make sense for different speakers to say, listeners report hearing different speakers. In experiment 2 we removed pragmatic information from the same stimuli by scrambling word order while leaving acoustic information intact. In contrast to experiment 1, results from the second experiment indicate no difference between our experimental conditions. We interpret these results as a top-down effect of pragmatic expectations: knowledge of conversational structure at least partially determines a listener’s perception of speaker changes in conversation.

## Introduction

Conversation is essential to human social life. It is through conversations that we establish and build societies, foster relationships, and cooperate. A critical skill in conversation is recognizing who is speaking and when a speaker switch occurs. Given that the same utterance can mean different things depending on who is speaking^1–3^, listeners’ ability to detect speaker changes in conversation is a fundamental prerequisite for successful communication.

In the absence of visual cues (e.g. while talking on the phone or listening to a podcast), listeners must identify speaker changes based on the auditory signal of the speakers’ voices. Humans are generally very good at this. In fact, the ability to recognize individuals from their vocalizations is an adaptive trait that many social animals demonstrate^4,5^. A specific aspect of human voice identification, however, is that listeners have access to both the *sound* of the talker as well as the *linguistic representations* that the utterance entails. That is, an utterances in conversation contains both a sound with acoustic information (e.g. frequency, amplitude, and pitch) as well as linguistic representations including phonetics, phonology, syntax, semantics, and pragmatics. How exactly these different sources of information interact in the process of speaker change detection is not clear from the existing literature.

Acoustic-focused accounts of speaker change detection suggest that listeners attend to bottom-up acoustic features in the auditory signal; when those features change, a speaker change is perceived. Kuwabara and Takagi^6^, for example, found that formant shifts significantly affect voice-individuality perception. Differences in vocal tract length also inform speaker identity perception^7^, with vocal tract features playing a more important role than glottal source features^8^. The reliability of acoustic features as cues for speaker change detection is, however, inconsistent. Evidence shows that voice information is not continuously monitored at a fine-grain level of acoustic representation^9,10^, and listeners can identify speakers even when these acoustic features are removed from the auditory signal^8,11^. Furthermore, an acoustic-focused account of speaker change detection does not sufficiently explain the phenomena of speaker change deafness, in which listeners fail to detect a speaker change^9^. These results suggest that bottom-up processing of acoustic information contained in the auditory speech signal alone does not suffice in explaining how listeners recognize when a speaker change occurs.

An alternative proposal is that bottom-up processing of acoustic features interacts with top-down linguistic representations in the process of speaker change detection. Listeners are better at recognizing who is speaking when they can access the phonological representations of the linguistic signal: listeners are more accurate at identifying who is speaking in their native language compared to an unfamiliar language^13^, and speaker identity recognition is compromised when abstract linguistic representations of words are impaired, such as in dyslexia^14^. In line with these findings, one study found that when listeners heard a story in both their native language and an unfamiliar language in which a speaker change occurred halfway through the story, they were better at detecting the speaker change in the unfamiliar language^15^. The authors explain this effect through listeners’ linguistic knowledge in their native language: when they have access to lexical and semantic representations and are not cued to listen for changes, their linguistic expertise overrides their detection of a change in acoustic features of the voice. This finding supports the idea that top-down linguistic knowledge shapes the way we direct attention and detect speaker changes in conversations^7,16^, aligning with a rich literature showing that speech processing involves top-down processes^17,18^.

While the aforementioned literature suggests that listeners draw on top-down linguistic representations to identify speakers, most of this research does not do justice to representing the rich communicative environment that humans converse in every day. Many of the studies cited here used only single vowels or consonant–vowel (CV) pairings in their stimuli, or asked participants listen to isolated sentences or stories to identify who is speaking and when a speaker switch occurs. Conversation, however, is characterized by rapid communicative interactions across multiple speakers and turns. Importantly, this involves processing at the pragmatic level of language representation: the knowledge of how language is used in social communication. To date, no studies that we know of have investigated how speaker change detection is influenced by top-down pragmatic expectations. Given that (1) conversations follow a pragmatic structure^19–21^, (2) pragmatics plays an important top-down role in comprehension^3^, and (3) speaker change detection is influenced by top-down processing at other levels of representation, we hypothesize that top-down pragmatic expectations bias listeners’ perception of speaker changes in conversation.

## Experiment 1

To investigate whether listeners’ pragmatic expectations influence their perception of who is speaking, we ran a randomized controlled experiment. We created a set of naturalistic conversational scenarios consisting of two turn construction units (TCUs)^22^, the smallest interactionally complete linguistic unit that can make up a turn in conversation. Both TCUs were spoken by the same speaker. The first TCU served as the context utterance, and the second TCU served as the target utterance. The experiment consisted of three experimental conditions. In the *congruent* condition, the second (target) utterance made sense for the same speaker to say, forming a plausible sequence of TCUs. In the *speaker violation* condition, the second (target) utterance did not make sense for the same speaker to say, but, importantly, would have formed a pragmatically coherent sequence of TCUs if it were spoken by a different speaker. In the *full violation* condition, the second (target) utterance formed a pragmatically incoherent sequence of TCUs regardless of whether it was spoken by the same speaker or different speakers. See Table 1 for examples. The task of the participants was to listen to the pairs of TCUs and indicate whether they heard the same speaker or different speakers. We hypothesize that listeners perceive a greater proportion of stimuli as spoken by different speakers in the *speaker violation* condition compared to both the *congruent* and *full violation* condition because the *speaker violation* condition is pragmatically more plausible when spoken by two different speakers. Because the *full violation* condition is pragmatically incoherent, we hypothesize that listeners rely on bottom-up acoustic features (as opposed to top-down pragmatic inferences) and thus perceive these stimuli as spoken by the same speaker. We therefore predict no difference between the *congruent* and *full violation* condition.

**Table 1:** Example stimulus from Experiment 1 in the congruent, speaker violation, and full violation conditions.

Context Utterance (TCU 1): “*We just moved into a new house.”* Target Utterance (TCU 2):			
	Congruent	Speaker violation	Full violation
Same speaker	“*Come by”*	“*Where?”*	“*You sure?”*

### Method

#### Participants

We report data from 60 participants who were recruited through the online platform Amazon Mechanical Turk; 20 men and 40 women. Participants’ ages ranged from 23 to 35 (*Median* = 31; *M* = 30.7; *SD* = 3.36). Originally, 68 participants were recruited, but 5 failed to complete the session and 3 failed one or more attention checks. All participants indicated having normal or corrected-to-normal vision and hearing and used headphones during the study. Participants were pre-screened to meet the following criteria: native English speaker, living in the USA, no history of psychiatric or neurological diagnoses, and no use of psychoactive medication within the preceding 6 months. All participants in this study provided written informed consent and were financially compensated for their time. Protocols were approved by the Tufts University Social, Behavioral, and Educational Research Institutional Review Board, and all research was performed in accordance with their guidelines and recommendations.

#### Stimuli

The stimulus materials consisted of audio recordings of conversations. Stimuli were recorded in soundproof rooms at a 44 kHz sampling rate using Shure MX153T/O-TQG Omnidirectional Earset Headworn Microphones. Six native speakers of American English (three men, three women) acted out the written conversational scenarios as naturally and informally as possible, without reading, to emulate natural conversation. All TCUs were recorded separately and processed using the software Praat^23^, version 6.0.48. The overall sound intensity of the recordings was normalized to 65 dB-SPL to prevent loudness differences between TCUs within the same stimulus and between stimuli. The relevant TCUs were then spliced together with a pause of 300 ms (a frequent gap between TCUs in natural conversation) in Python^24^, using the package Parselmouth, version 0.4.1^25^. Both TCUs in each stimulus were always spoken by the same speaker. The average length of the context TCUs was 1.71 s, while the average length of the target TCUs was 0.52 s. The average length of the stimuli (both TCUs separated by 300 ms of silence) was 2.53 s. A total of 375 stimuli were created, with 125 items per conditions. Each target utterance was fully counterbalanced across conditions: the exact same second TCU appeared in each of the three conditions. The stimuli were then divided into three experimental lists such that no TCUs were repeated. Each list contained an approximately equal proportion of items per condition (*congruent*, *speaker violation* and *full violation*): 42 stimuli from two of the conditions, and 41 stimuli from the remaining condition. The condition with one less stimulus item varied between the three lists. Similarly, the six recorded speakers appeared as equally as possible in each condition and within each list. The order of the stimuli was randomized.

#### Procedure

The study was conducted online via the Qualtrics platform^26^. Participants completed a pre-screening survey to ensure that they met the eligibility criteria to participate in the study. They were instructed to listen carefully to the conversations and to indicate whether they heard the same speaker or different speakers in the first and second TCU by selecting either “same” or “different” from a multiple-choice menu using a mouse click after hearing the stimuli. Stimuli from the three conditions were presented in a randomized order. Prior to carrying out the task, participants completed a guided practice with examples to familiarize themselves with the task. Five “catch” trials were included at random intervals to ensure that participants were paying attention throughout the experiment and to identify and omit data from bots. These trials consisted of audio recordings of a speaker instructing the participant to either select “same” or “different” from the two possible response options. Participants listened to a total of 125 conversations and took approximately 20 min to complete the study.

#### Statistical analysis

We analyzed the data with logistic linear mixed-effects regression models^27^ with R version 4.11^28^. We performed both a Bayesian and a traditional frequentist analysis. Bayesian analyses provide information about the strength of the evidence in favor of either the alternative or the null hypothesis, while frequentist tests compute the probability of the data or more extreme data under a null hypothesis. For the Bayesian analyses, we report Bayes factors, representing the relative probability of the observed data under model *a* over model *b*, to determine the model under which the data are the most likely. In this paper, we interpret Bayes factor values using evidence categories from Wetzels^29^ adapted from Jeffreys^30^.

For the Bayesian models, we used glmer from the rstan^31^, and rstanarm^32^ packages, and then used the bridgesampling package^33^ to obtain Bayes factors for model comparisons. The rstanarm package estimates multilevel models using full Bayesian Inference via Markov Chain Motel Carlo (MCMC) estimation. In the current analysis, we used the default (weakly informative) priors from rstan. We added our fixed effect (condition) and random effects (participants and items) incrementally to a minimal model and tested if the inclusion of the additional term was justified by comparing the likelihood of the data under both models.

For the frequentist models, we used glmer from the R package lme4^34^ and used the lmerTest package^35^ to calculate p-values for models. Again, we added the same fixed and random effects incrementally to a minimal model and then used the likelihood ratio test for model comparison to test if the inclusion of an additional term was justified^36^.

### Results and discussion

**Figure 1:**
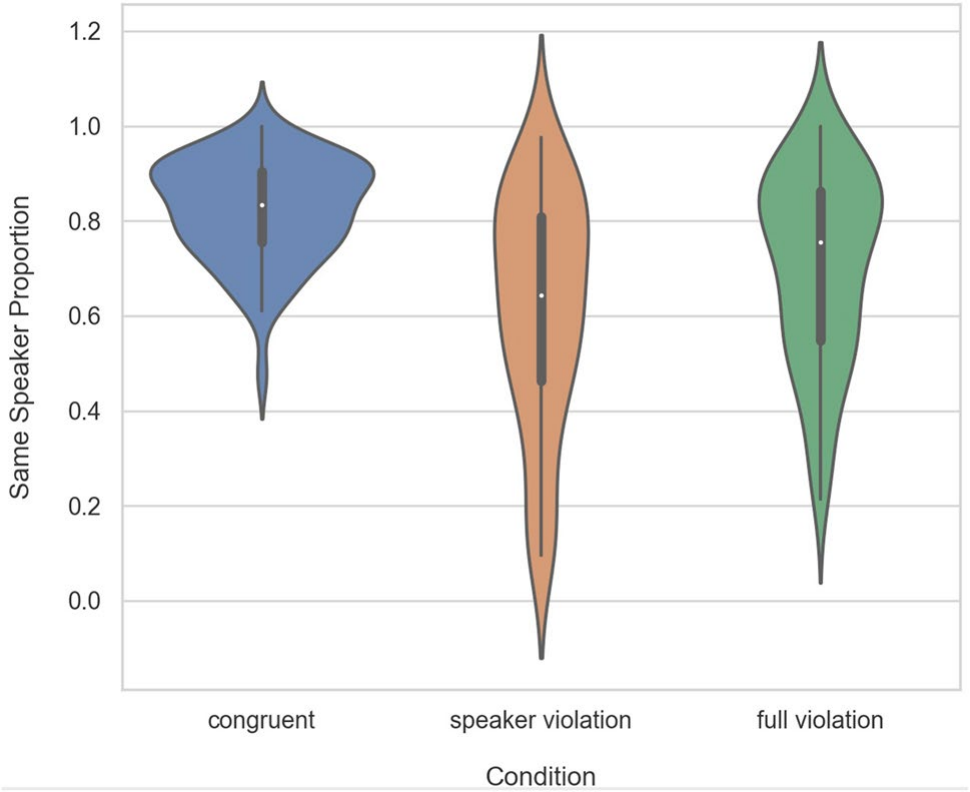
Violin plot showing the proportion of stimuli perceived as spoken by the same speaker in the *congruent*, *speaker violation* and *full violation* conditions in Experiment 1. The white dot indicates the median, the thick gray line represents the interquartile range, and the thin gray line represents the rest of the distribution barring outliers. The overall shape indicates the kernel density estimation of the underlying distribution.

In the congruent condition, 82.44% of trials were perceived as being spoken by the same speaker. In the speaker violation condition, only 61.4% of trials were heard as the same speaker, and in the full violation trials, 70.6% of trials were heard as the same speaker. The data are visualized in Fig. 1. The distribution of participants’ responses across the 6 speakers of the stimuli are reported in Table 2.

**Table 2:** Frequencies of speaker ratings in Experiment 1 separated by speakers of the stimuli.

	Recorded speakers					
	1	2	3	4	5	6
Same speaker	0.70	0.66	0.75	0.74	0.59	0.86
Different speaker	0.30	0.34	0.25	0.26	0.41	0.14

According to the Bayesian analysis, the model under which the data were most likely was one that contained condition as a fixed factor, and random intercepts for both participants and items. The Bayes factor for the data under this model was 6.23 × 10^74^, providing decisive evidence for this model over the null model (intercept only). This indicates a main effect of condition: whether participants heard the same or different speakers was affected by our experimental conditions.

The summary for the final model for the data is shown in Table 3, with *congruent* as the reference level. Participants heard the second TCU in the *congruent* stimuli as the same speaker more often than in both the *speaker violation* and the *full violation* condition, with a model estimated probability of 1. Participants also heard the second TCU in the *speaker violation* condition as different speakers more often than in the *full violation* condition, with an estimated model probability of 1.

**Table 3:** Summary of Bayesian Logistic Linear Mixed Effects Model for Experiment 1 with congruent as the reference level.

Fixed effects	Mean	95% CI	P(b > 0)	P(b < 0)
(Intercept)	− 2.05	− 2.40 to − 1.71	0	1
Speaker violation	1.41	1.26–1.56	1	0
Full violation	0.87	0.71–1.02	1	0

Mean represents the posterior mean unstandardized beta coefficient, 95% CI represents the credible interval around the mean, P(b > 0) represents the probability that the coefficient is greater than zero, and P(b < 0) represents the probability that the coefficient is less than zero.

The frequentist analysis was consistent with these findings. The final model with *congruent* as the reference level is shown in Table 4, and with *full violation* as the reference level in Table 5, and contains condition as a fixed factor with random intercepts for participants and items. We found a significant main effect of condition (*X*^*2*^(2) = 335.58, *p* < 0.001). Participants heard different speakers more frequently in both the *speaker violation* (β_speaker violation_ = 1.41, *p* < 0.001) and the *full violation* (β_full violation_ = 0.87, *p* < 0.001) condition compared to the *congruent* condition. Participants also heard different speakers more frequently in the *speaker violation* condition compared to the *full violation* condition (β_speaker violation_ = 0.55, *p* < 0.001).

**Table 4:** Summary of Frequentist Logistic Linear Mixed Effects Model for Experiment 1 with congruent as the reference level.

Fixed effects	Estimate	Std. error	Z value	Pr( >|z|)
(Intercept)	− 2.05	0.17	− 12.00	** < 0.001**
Speaker violation	1.41	0.08	18.32	** < 0.001**
Full violation	0.87	0.08	11.14	** < 0.001**

Significant values are in bold.

**Table 5:** Summary of Frequentist Logistic Linear Mixed Effects Model for Experiment 1 with full violation as the reference level.

Fixed effects	Estimate	Std. error	Z value	Pr( >|z|)
(Intercept)	− 1.19	0.17	− 7.10	**< 0.001**
Congruent	− 0.87	0.08	− 11.14	** < 0.001**
Speaker violation	0.55	0.07	7.89	** < 0.001**

Significant values are in bold.

The aim of this study was to investigate whether listeners’ pragmatic expectations in conversation have a top-down influence on their perception of who is speaking. Our data confirm our first hypothesis: when two segments in conversation make more sense for different speakers to say, participants report hearing different speakers, even in the absence of a speaker change. We explain this as a top-down effect of pragmatic representation: listeners draw on their knowledge of normative conversational structure such that when an utterance makes sense for a different speaker to say, they infer that a speaker change occurred and perceive a different voice. Interestingly, participants also reported hearing different speakers more frequently when the two segments did not make sense for a different speaker *or* the same speaker to utter. Our hypothesis that listeners draw more strongly on bottom-up acoustic features when an utterance is pragmatically incoherent and unpredictable was therefore not supported. We also explain this result as an effect of pragmatic inference: incoherent adjacent turns are more likely to be uttered by two different speakers than by the same speaker.

A surprising result of Experiment 1 is that in the *congruent* condition, only 82% of the stimuli were perceived as spoken by the same speaker. This is unexpected because both the acoustic features as well as the pragmatic context should reinforce the coherence of these stimuli, i.e. we would expect 100% to be perceived as spoken by the same speaker. This finding could be driven by several factors. First, participants in web-based experiments tend to be less attentive than those participating in in-person experiments^37^, so our result could be explained through noise in the data.^.^ However, “catch questions” and large sample sizes such as ours should reduce and compensate for this noise^38^. Second, our experimental design could have influenced participants’ selection of answers. Given that all our stimuli were always spoken by the same speaker but that participants are, in general, not inclined to keep giving the same answer, participants could have been biased to select having heard different speakers. Since our effect of condition is very large, this possibility does not alter our conclusion: the pragmatic relationship between two TCUs plays a role in speaker change detection. The potential of our experimental design biasing participants’ answers does, however, suggest that this effect may be more subtle in real, everyday conversations.

Though our results strongly implicate pragmatic context in speaker change detection, our experimental design does not allow us so strictly rule out any influence of acoustic cues. Research suggests a strong interaction between top-down lexical and sentential content and indexical (talker-specific) content^39,40^: the presence of linguistic context at the word and sentence levels affects participants’ discrimination of voice cues. In order to test the causal role of pragmatic context in speaker change detection and to further rule out any effect of acoustic cues in Experiment 1, we ran a follow up experiment in which we removed all pragmatic information from the same stimuli, leaving acoustic information intact.

## Experiment 2

In Experiment 1, participants listened to utterances in conversation that were either pragmatically coherent or incoherent, and our results suggest that pragmatic coherence influences listeners’ speaker change detection. To investigate the causal nature of this top-down pragmatic effect on speaker change detection, we ran a control experiment in which we took the same conversational stimuli and removed all pragmatic information while preserving the acoustic information. In our second experiment, we tested this by asking a new set of participants to partake in the same task using the same stimuli with the exception that the word order in the stimuli was scrambled, removing any pragmatic information from the utterances. We hypothesized that the rate of speaker change detection would now be identical across conditions.

### Method

#### Participants

Sixty participants were recruited from Amazon Mechanical Turk, including 33 men and 27 women whose ages ranged from 23 to 35 (*Median* = 32; *M* = 31.15; *SD* = 3.43). Participants were compensated and screened according to the same procedures are Experiment 1. All participants in this study provided written informed consent and were financially compensated for their time. Protocols were approved by the Tufts University Social, Behavioral, and Educational Research Institutional Review Board, and all research was performed in accordance with their guidelines and recommendations. None of the participants in this experiment had participated in Experiment 1.

#### Stimuli and procedure

To remove the pragmatic information from the stimuli, we randomly shuffled the order of the words. For each TCU of the stimulus, we first found the boundary between each word using timing information from Gailbot^41^, an automated transcription software that calculates word-by-word timing. All word boundaries were then manually inspected and corrected and moved to zero crossings in the waveform using Praat^20^. Sound files were cut at the word boundaries and spliced back together in a random order. Because randomizing the word order made it difficult to recognize the boundary between TCUs, and our task asked participants if they hear the same or different speakers in the first and second TCU, we included a beep sound between the two TCUs. The beep was 300 ms long, the same length as the gap between turns in Experiment 1. To ensure that no pragmatic information was retained in the stimuli, no more than two words from the original word order in Experiment 1 were adjacent to each other, and all stimuli were manually checked to confirm unintelligibility. See Table 6 for example stimuli.

**Table 6:** Example stimulus from Experiment 2 in the congruent, speaker violation, and full violation conditions.

Context Utterance (TCU 1): “*moved we new just a into house”* Target Utterance (TCU 2):			
	Congruent	Speaker violation	Full violation
Same speaker	“*by come”*	“w*here”*	“*sure you”*

The experimental procedure was identical to that of Experiment 1. Participants were instructed to listen to the scrambled conversation segments and to indicate whether they heard the same or different speakers before and after the beep.

### Results and discussion

**Figure 2:**
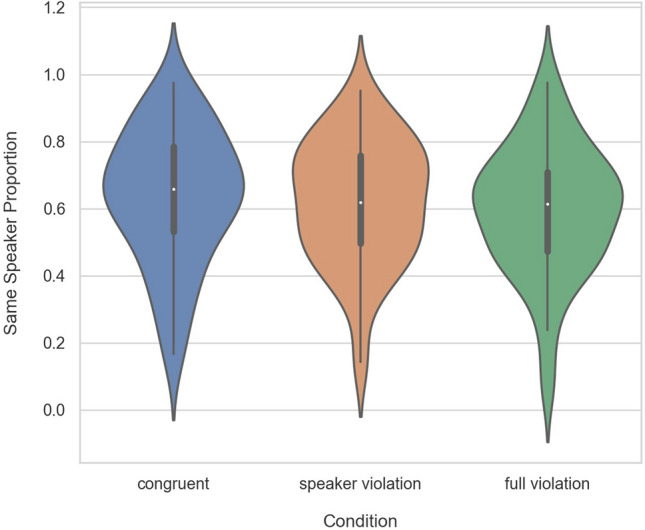
Violin plot showing the proportion of stimuli perceived as spoken by the same speaker in the *congruent*, *speaker violation* and *full violation* conditions in Experiment 2. The white dot indicates the median, the thick gray line represents the interquartile range, and the thin gray line represents the rest of the distribution barring outliers. The overall shape indicates the kernel density estimation of the underlying distribution.

We analyzed the data following the same procedures as Experiment 1. In the congruent condition, 62.24% of trials were perceived as being spoken by the same speaker. In the speaker violation condition, only 62.2% of trials were heard as the same speaker, and in the full violation trials, 60.5% of trials were heard as the same speaker. The distribution of participants’ responses across the 6 speakers of the stimuli are reported in Table 7. As Fig. 2 confirms, there was no observable difference in speaker ratings between our three experimental conditions when the pragmatic information was removed from the stimuli. Both the Bayesian and frequentist analyses confirm this finding. According to the Bayesian analysis, the model under which the data were most likely was one that contained only random intercepts for both participants and items. The data were 1938 times more likely under this null model compared to a model that also contained condition as a fixed effect, indicating that there was no main effect of condition. For the frequentist model, the final model was the same. There was no main effect of condition (*X*^*2*^(2) = 2.82, *p* = 0.24), and none of the conditions significantly predicted whether participants heard the same or different speakers. See Table 8 for the model with *congruent* as the reference level and Table 9 for the model with *full violation* as the reference level. The results from the current study thus strongly indicate that the effect found in Experiment 1 can be explained by pragmatic context rather than acoustic features in the auditory signal, which remained, for the most part, identical across experiments. We discuss implications and possible limitations to this conclusion below.

**Table 7:** Frequencies of speaker ratings in Experiment 2 separated by speakers of the stimuli.

	Recorded speakers					
	1	2	3	4	5	6
Same speaker	0.57	0.63	0.57	0.63	0.58	0.73
Different speaker	0.43	0.37	0.43	0.37	0.42	0.27

**Table 8:** Summary of Frequentist Logistic Linear Mixed Effects Model for Experiment 2 with congruent as the reference level.

Fixed effects	Estimate	Std. error	Z value	Pr( >|z|)
(Intercept)	− 0.61	0.14	− 4.49	** < 0.001**
Speaker violation	0.01	0.05	0.102	0.92
Full violation	0.09	0.06	1.50	0.13

Significant values are in bold.

**Table 9:** Summary of Frequentist Logistic Linear Mixed Effects Model for Experiment 2 with full violation as the reference level.

Fixed effects	Estimate	Std. error	Z value	Pr( >|z|)
(Intercept)	− 0.52	0.14	− 3.80	** < 0.001**
Congruent	−0.010	0.06	− 1.50	0.13
Speaker violation	− 0.09	0.06	− 1.40	0.16

Significant values are in bold.

## General discussion

Our experiments demonstrate that listeners use their expectations of dialogue coherence to infer who is speaking in conversation. We find that when an utterance makes sense for a different speaker to say, listeners experience the illusion that a speaker change has occurred and hear a different voice, even in the absence of a speaker change. Pragmatic knowledge thus has a top-down influence on how speaker identity is perceived by listeners in conversation.

Our study is experimentally well-controlled, supporting our conclusions, and has a high level of ecological relevance: speaker change detection is a process that almost all of us engage in every day. That said, there are several limitations that warrant discussion. First, some of the experimental manipulations in Experiment 2 that were designed to remove pragmatic intelligibility may also have altered acoustic features that could be relevant to speaker identification. Specifically, scrambling the order of the words impacts the rhythmic structure underpinning the speech in the utterance. While the literature on the role of speech rhythm in speaker change detection is limited, research suggests consistent between-speaker variability of speech rhythm^42^, which, according to one study, contributes to speaker recognition^43^. The acoustic features of signal may therefore not have been identical across Experiments 1 and 2. However, the acoustic voice cues that have been shown to be most salient for the discrimination between voices of different talkers—fundamental frequency (F0)^39,44–46^ and vocal tract length^47^—were not altered by Experiment 2. The long term average spectrum^48^, another cue used by listeners to differentiate between voices^49^, also remained consistent across our experiments.

Further research is needed to understand the effect of scrambled word order on acoustic features that are relevant for talker discrimination.

Second, it is possible that acoustic features in the signal were attended to differently across Experiments 1 and 2, which may have influenced participants’ auditory perceptions. Research shows top-down effects of linguistic content on the differentiation of voice cues that are relevant to speaker differentiation. The presence of linguistic content at the word level, for example, increases participants’ ability to differentiate between vocal tract lengths of voices^39^, and both vocal tract length and F0 differentiation increases in the context of sentences^50^. These findings indicate a top-down effect of linguistic content on how we integrate acoustic information in speaker voice discrimination processes, meaning that participants in our experiment may have actually been more sensitive to differences in acoustic features in Experiment 1 compared to Experiment 2. While this insight does not alter our conclusions, it does highlight a possible difference in acoustic feature processing between our two experiments.

A third possibly significant difference between our experiments is the presence of the beep tone separating the TCUs in Experiment 2, which could have disturbed the evaluation of the voices. The beep could be acting as a masking stimulus, affecting and compromising the perception of the speech^51^ and rendering it more difficult to compare the speaker voices of the first and second TCU in Experiment 2 compared to Experiment 1. The literature suggests that the ability to detect information in the speech stream is reduced when attention is directed away from speech^52^ and work on change deafness specifically indicates that distractors and the way in which we direct attention have robust effects on change detection^9,12^. The presence of the beep in Experiment 2 thus reduces the comparability of our two experiments.

Our primary interest in the current study was the difference in the effect of condition between experiments 1 and 2, which shows that pragmatic information influences listeners’ perception of when a speaker change occurs. It is, however, also worth comparing the raw proportions of speaker perceptions between the two experiments. In experiment 2 listeners perceived the same speaker in approximately 62% of the stimuli across all three conditions. Given that both TCUs were spoken by the same speaker in all stimuli and that no pragmatic information was available in this experiment, it is surprising that participants reported hearing such a large number of different speakers. One possible explanation for this finding is that our binary choice experimental design biased speakers to select hearing different speakers more than they actually did. Another possibility is that the beep tone, as discussed above, had a masking effect, reducing the comparability of the voices in the two TCUs. Paired with our experimental design, listeners may have therefore been swayed to perceive two different speakers more than if there had been no beep tone. Finally, it is possible that the destruction of the rhythmic structure of the utterances in Experiment 2, which, as discussed above, may be a possible cue for speaker change detection, affected participants’ perception of speaker changes. It is particularly interesting that the response rate in all conditions of Experiment 2 is identical to that of the *speaker violation* condition in Experiment 1, indicating that the high proportion of “different speaker” responses may be an artifact of our experimental design rather than any acoustic processing differences across the two experiments.

Though the limitations discussed above impact the comparability of experiments 1 and 2 and should be considered in future research on speaker change detection, we do not think that they significantly alter our conclusion that listeners use their pragmatic expectations of dialogue coherence to infer who is speaking in conversation. We therefore now turn to the practical and theoretical implications of this finding. First, our work provides important insight for computational models of language processing. Automatic analysis of conversation audio remains a challenge with multiple talkers, intonational variation and overlapping speech. The results from this study provide further evidence that speaker change detection relies on linguistic representations at multiple levels, including the pragmatic level. Understanding the cognitive basis of how humans detect speaker changes in conversation is fundamental to implementing this process in machines.

Second, and most importantly, the findings from this study have important theoretical implications. They show that prior talk in conversation contains a rich structure that proactively affects interpretations of something as low-level as acoustic information. This aligns with a rich literature across many modalities demonstrating top-down effects of cognition on the interpretation of a percept. Furthermore, our results suggests that listeners assume cooperativity in conversation: when an utterance makes more sense for one speaker to say than another, listeners hear the speaker that makes the most sense. Given that the world is noisy and the bottom-up signal is often unreliable, we suggest that this top-down mechanisms of speaker change detection has evolved for optimal social communication.

## Data Availability

The datasets generated during and analyzed during the current study are available in the Open Science Framework at https://osf.io/s3yve/.

## References

[1] Krauss RM, Fussell SR (1991). Perspective-taking in communication: Representations of others’ knowledge in reference. Soc. Cogn..

[2] Metzing C, Brennan SE (2003). When conceptual pacts are broken: Partner-specific effects on the comprehension of referring expressions. J. Mem. Lang..

[3] Van Berkum JJA, Van Den Brink D, Tesink CMJY, Kos M, Hagoort P (2008). The neural integration of speaker and message. J. Cogn. Neurosci..

[4] Wiley RH (2005). Individuality in songs of Acadian flycatchers and recognition of neighbours. Anim. Behav..

[5] Balcombe JP, McCracken GF (1992). Vocal recognition in mexican free-tailed bats: Do pups recognize mothers?. Anim. Behav..

[6] Kuwabara H, Takagi T (1991). Acoustic parameters of voice individuality and voice-quality control by analysis-synthesis method. Speech Commun..

[7] Gaudrain, E., Li, S., Shen Ban, V. & Patterson, R. D. The role of glottal pulse rate and vocal tract length in the perception of speaker identity. *Interspeech 2009* (2009).

[8] Lavner Y, Gath I, Rosenhouse J (2000). The effects of acoustic modifications on the identification of familiar voices speaking isolated vowels. Speech Commun..

[9] Fenn KM (2011). When less is heard than meets the ear: Change deafness in a telephone conversation. Q. J. Exp. Psychol..

[10] Sell G, Suied C, Elhilali M, Shamma S (2015). Perceptual susceptibility to acoustic manipulations in speaker discrimination. J. Acoust. Soc. Am..

[11] Sheffert SM, Pisoni DB, Fellowes JM, Remez RE (2002). Learning to recognize talkers from natural, sinewave, and reversed speech samples. J. Exp. Psychol. Hum. Percept. Perform..

[12] Vitevitch MS (2003). Change deafness: The inability to detect changes between two voices. J. Exp. Psychol. Hum. Percept. Perform. Wash..

[13] Perrachione TK, Wong PCM (2007). Learning to recognize speakers of a non-native language: Implications for the functional organization of human auditory cortex. Neuropsychologia.

[14] Perrachione TK, Del Tufo SN, Gabrieli JDE (2011). Human voice recognition depends on language ability. Science.

[15] Neuhoff JG, Schott SA, Kropf AJ, Neuhoff EM (2014). Familiarity, expertise, and change detection: Change deafness is worse in your native language. Perception.

[16] Clarke J, Gaudrain E, Chatterjee M, Başkent D (2014). T’ain’t the way you say it, it’s what you say: Perceptual continuity of voice and top–down restoration of speech. Hear. Res..

[17] Ganong WF (1980). Phonetic categorization in auditory word perception. J. Exp. Psychol. Hum. Percept. Perform..

[18] Norris D, McQueen JM, Cutler A (2003). Perceptual learning in speech. Cognit. Psychol..

[19] Levinson SC (1983). Conversational structure. Pragmatics.

[20] Schegloff EA (2007). Sequence Organization in Interaction: A Primer in Conversation Analysis I.

[21] Schegloff EA, Sacks H (1973). Opening up closings. Semiotica.

[22] Sacks H, Schegloff EA, Jefferson G (1974). A simplest systematics for the organization of turn-taking for conversation. Language.

[23] Boersma, P. & Weenink, D. *Praat: Doing Phonetics by Computer* (2019).

[24] Van Rossum, G. & Fred, D. *Python 3 Reference Manual*. (CreateSpace, 2009).

[25] Jadoul Y, Thompson B, de Boer B (2018). Introducing Parselmouth: A Python interface to Praat. J. Phon..

[26] Qualtrics. (2022).

[27] Baayen RH, Davidson DJ, Bates DM (2008). Mixed-effects modeling with crossed random effects for subjects and items. J. Mem. Lang..

[28] R Core Team (2021). R: A Language and Environment for Statistical Computing.

[29] Wetzels R (2011). Statistical evidence in experimental psychology: An empirical comparison using 855 t tests. Perspect. Psychol. Sci..

[30] Jeffreys H (1961). Theory of Probability.

[31] Stan Development Team. *RStan: The R interface to Stan*. (2020).

[32] Goodrich B, Gabry J, Ali I, Brilleman S (2020). rstanarm: Bayesian applied regression modeling via Stan. R Package.

[33] Gronau QF, Singmann H, Wagenmakers E-J (2020). bridgesampling: An R package for estimating normalizing constants. J. Stat. Softw..

[34] Bates D, Mächler M, Bolker B, Walker S (2015). Fitting linear mixed-effects models using lme4. J. Stat. Softw..

[35] Kuznetsova A, Brockhoff PB, Christensen RHB (2017). lmerTest package: Tests in linear mixed effects models. J. Stat. Softw..

[36] Pinheiro JC, Bates DM (2000). Linear Mixed-Effects Models: Basic Concepts and Examples Mixed-Effects Models in S and S-PLUS.

[37] Oppenheimer DM, Meyvis T, Davidenko N (2009). Instructional manipulation checks: Detecting satisficing to increase statistical power. J. Exp. Soc. Psychol..

[38] Paolacci G, Chandler J, Ipeirotis PG (2010). Running experiments on Amazon Mechanical Turk. Judgm. Decis. Mak..

[39] Koelewijn T, Gaudrain E, Tamati T, Başkent D (2021). The effects of lexical content, acoustic and linguistic variability, and vocoding on voice cue perception. J. Acoust. Soc. Am..

[40] Zaltz Y, Goldsworthy RL, Kishon-Rabin L, Eisenberg LS (2018). Voice discrimination by adults with cochlear implants: The benefits of early implantation for vocal-tract length perception. J. Assoc. Res. Otolaryngol..

[41] Umair M, Mertens JB, Albert S, Ruiter JP (2022). de GailBot: An automatic transcription system for Conversation Analysis. Dialogue Discourse.

[42] Dellwo V, Leemann A, Kolly M-J (2015). Rhythmic variability between speakers: Articulatory, prosodic, and linguistic factors. J. Acoust. Soc. Am..

[43] Van Dommelen WA (1987). The contribution of speech rhythm and pitch to speaker recognition. Lang. Speech.

[44] Darwin CJ, Brungart DS, Simpson BD (2003). Effects of fundamental frequency and vocal-tract length changes on attention to one of two simultaneous talkers. J. Acoust. Soc. Am..

[45] Baumann O, Belin ÆP, Baumann O, Belin P, Baumann O (2010). Perceptual scaling of voice identity: Common dimensions for different vowels and speakers. Psychol. Res..

[46] Chhabra S, Badcock JC, Maybery MT, Leung D (2012). Voice identity discrimination in schizophrenia. Neuropsychologia.

[47] Hillenbrand J, Getty LA, Wheeler K, Clark MJ (1994). Acoustic characteristics of American English vowels. J. Acoust. Soc. Am..

[48] Löfqvist A (1986). The long-time-average spectrum as a tool in voice research. J. Phon..

[49] Durlach N (2006). Auditory masking: Need for improved conceptual structure. J. Acoust. Soc. Am..

[50] Sinnett S, Costa A, Soto-Faraco S (2006). Manipulating inattentional blindness within and across sensory modalities. Q. J. Exp. Psychol..

[51] Eramudugolla R, Irvine DRF, McAnally KI, Martin RL, Mattingley JB (2005). Directed attention eliminates ‘change deafness’ in complex auditory scenes. Curr. Biol..

[52] Neuhoff JG, Bochtler KS (2018). Change deafness, dual-task performance, and domain-specific expertise. Q. J. Exp. Psychol..

